# Education and Meat Consumption and Reduction: The Mediating Role of Climate Literacy

**DOI:** 10.3390/foods14193333

**Published:** 2025-09-25

**Authors:** Andrej Kirbiš, Stefani Branilović

**Affiliations:** Department of Sociology, Faculty of Arts, University of Maribor, 2000 Maribor, Slovenia

**Keywords:** meat consumption, sustainability, education, climate literacy, pro-environmental behaviour

## Abstract

Meat consumption, a key factor in both environmental sustainability and public health, is strongly influenced by educational characteristics, with higher levels of education often associated with more sustainable dietary patterns. However, research examining the mechanisms through which education influences meat-related behaviours remains limited. This study investigates the mediating role of climate literacy in the relationship between educational characteristics and meat consumption patterns among adults and school-enrolled youth in Slovenia. We used survey data from a sample of 2990 individuals (aged 14–88) to examine how educational stage, track, and level impact meat consumption and reduction. Our focus was on climate literacy as a multidimensional construct, comprising climate knowledge, attitudes, and pro-environmental behaviour. The findings indicate that young people in the tertiary educational track tend to eat less meat, have already reduced their meat consumption in the past, and intend to further reduce it in the future, compared to secondary track students, with climate attitudes playing a mediating role in all three cases. For adults, a tertiary educational level, relative to a secondary level, was linked to lower meat consumption, an association largely explained by more positive climate attitudes. By contrast, adults with only primary education consume meat more often and are less inclined to cut back in the future. Among secondary school students, both vocational and general school groups reported greater past and intended meat reductions than their peers in professional schools. The findings underscore the importance of integrating climate literacy, especially fostering pro-climate attitudes, into educational programmes to promote sustainable dietary choices.

## 1. Introduction

Pro-environmental behaviour refers to actions that reduce environmental harm and contribute positively to ecological sustainability, such as minimising greenhouse gas emissions and conserving natural resources [1,2]. Common behaviours in this domain include recycling [3,4], using public transport [5], managing waste efficiently [6,7], conserving energy [8], and purchasing environmentally friendly products [1]. Reducing meat consumption is increasingly recognised as a pro-environmental behaviour, as animal-based food production has a disproportionately high environmental impact compared to plant-based alternatives [9,10]. Specifically, meat production contributes approximately 30% of global greenhouse gas emissions and involves substantial energy, land, and water use [11,12]. Beyond environmental harm, high intake of red and processed meat is associated with increased health risks, including cardiovascular diseases, diabetes, obesity, and cancer [11,13]. As a result, global health and environmental authorities have emphasised the transition to sustainable diets, defined as those with low environmental impact and positive health outcomes [9,13,14].

Previous studies on meat intake and meat reduction have largely focused on demographic and socioeconomic predictors [15,16,17], on environmental knowledge, attitudes, and lifestyles [18,19,20]. These studies consistently show that lower meat consumption is associated with being female [21,22], younger [16,23], and urban-based [24]. The research also shows that those with higher education [25,26,27] and more climate-literate [28,29] behave in an environmentally friendly way.

However, the potential mediating role of climate literacy in the relationship between education and pro-environmental behaviour, especially meat consumption, remains underexplored. While higher education is typically linked to greater environmental concern and action [30,31,32], climate literacy, particularly its domains of knowledge, concern, and behaviour, may explain this link more precisely [33,34,35].

The present study investigates whether the educational level among adults, the educational stage among youths, and the educational track among secondary-school students predict meat intake and intentions to reduce meat consumption, and whether climate literacy mediates these relationships. In particular, we examine climate literacy as a multidimensional construct, comprising knowledge, attitudes, and climate-related behaviour, to assess which dimension most strongly mediates the link between educational characteristics and meat-related behaviours.

## 2. Theoretical Background

### 2.1. Education and Pro-Environmental Behaviour

Education is frequently cited as a key predictor of pro-environmental behaviour. Higher educational attainment tends to correlate with increased environmental awareness and greater adoption of sustainable practices, including the purchase of organic products [36], energy-saving behaviours [37], and recycling [38].

Numerous studies indicate that highly educated individuals are more likely to reduce meat consumption or abstain altogether, compared to those with lower educational levels [17,39,40]. A multi-country study across Europe found that higher education levels were associated with lower intake of processed meat [31], while Klink et al. [30] showed that highly educated individuals consume less meat overall.

Similar patterns were observed in the U.S., Germany, and Australia [41,42]. Alles and colleagues [16] also found that in comparison to less-educated individuals, the highly educated are more likely to be vegetarians than meat-eaters (see also Lehto et al. [43]). In sum, educational attainment is a consistent predictor of meat-related behaviours and broader ecological awareness.

### 2.2. Climate Literacy as a Mediator

Climate literacy encompasses knowledge, skills, and attitudes that empower individuals to adopt climate- and environmentally friendly behaviours [34,44]. Empirical evidence shows that individuals with higher climate literacy are more likely to perceive climate change as a pressing issue and act accordingly, especially when they believe their actions can make a difference [45].

Several studies support the link between environmental knowledge and pro-environmental attitudes and behaviours [19,39,46]. For instance, Pan et al. [32] found that climate literacy significantly increased environmental concern in China, reinforcing that climate knowledge is a fundamental driver of environmental action [34]. For example, individuals with greater environmental knowledge are more likely to engage in behaviours such as saving energy [47].

Additionally, values such as environmental commitment and lifestyle choices are associated with sustainable behaviours [18]. Awareness of the negative environmental impact of meat consumption has also grown, and individuals who believe that meat consumption negatively influences climate change are more likely to reduce their meat intake [35]. Therefore, environmental concern is a key motivator for meat reduction [24].

Importantly, education enhances climate literacy, as shown by cross-national studies [48,49]. However, most of this research focuses on educational interventions and their outcomes rather than general education levels. For instance, Choi et al. [50] reported increased climate literacy among junior high school students following an SSI-STEAM intervention. Similar improvements were observed in Spain [51] and in a study involving 245 children aged 9 to 13 [52].

In summary, education contributes to pro-environmental behaviour indirectly through enhancing climate literacy. According to the knowledge-concern-action model, more years of education increase environmental knowledge, which in turn fosters environmental attitudes and behaviours [19]. Mata et al. [31] showed that environmental attitudes mediate the link between education and processed meat consumption. Davitt et al. [28] and Borusiak et al. [29] demonstrated that environmental attitudes and awareness of meat’s climate impact predict plant-based dietary behaviours.

### 2.3. Literature Gap

Although prior studies have consistently shown that higher levels of education are associated with lower meat consumption and greater climate literacy, the mechanisms linking these relationships remain insufficiently explored. In particular, most existing research has considered education and climate literacy as separate predictors of pro-environmental behaviour [26,33], rather than examining whether climate literacy mediates the effect of education on behaviours such as meat intake and reduction intentions.

Moreover, while some studies have investigated the role of climate change knowledge or environmental concern [32,34], few existing studies treated climate literacy as a multidimensional construct that integrates knowledge, attitudes, and climate-related behaviour [45,46]. In fact, as a recent systematic review on climate literacy measurement shows, only a minority of studies apply a comprehensive, three-dimensional approach that includes knowledge, attitudes, and actual climate-related behaviour [53]. The majority of studies focus solely on the knowledge dimension or combine only two of the three dimensions [54,55,56], rarely capturing what individuals know, feel, and actually do regarding climate issues. This limited scope constrains our ability to understand the full mediating role of climate literacy in shaping behavioural outcomes such as meat consumption and meat reduction.

To our knowledge, no study has systematically examined whether all three dimensions of climate literacy mediate the relationship between educational level, stage, and track, and meat intake and meat reduction patterns, neither among adults nor youth populations. By applying a comprehensive climate literacy framework and testing these mediation pathways, our study addresses these critical empirical and theoretical gaps.

### 2.4. Study Aim and Hypotheses

Building on the theoretical framework and identified literature gaps, this study examines how educational level among adults and educational stage and track among youth influence meat-related behaviours, and whether climate literacy mediates these relationships. Specifically, we explore the mediating role of climate literacy across its three dimensions, knowledge, attitudes, and behaviour, in order to understand the pathways through which education may shape dietary sustainability.

From this, we derive the following hypotheses:

**H1.**  *Individuals with higher educational attainment (a), educational stage (b) and educational track (c) report lower levels of meat consumption and stronger intentions to reduce meat intake.*

**H2.**  *Climate literacy mediates the relationship between educational level, stage and track and meat-related behaviours, such that higher educational characteristics are associated with greater climate literacy, which in turn predicts reduced meat consumption and stronger reduction intentions.*

In addition, we pose the following research question (RQ1):

**RQ1.**  *Which of the three dimensions of climate literacy (knowledge, attitudes, or behaviour) serve as the strongest mediators in the relationship between education (level, stage and track) and meat-related behaviours?*

## 3. Method

### 3.1. Sample

In this study, we analysed survey data from a sample of Slovenian people (*n* = 2990, 14–88 years, M_(age)_ = 29.98, SD_(age)_ = 17.41, 54.2% women), which was initially collected to examine climate literacy levels among both young people and adults in Slovenia. The full sample included primary school students (*n* = 405), secondary school students (*n* = 799), tertiary students (*n* = 741), employed individuals (*n* = 580), unemployed individuals (*n* = 122), self-employed individuals (*n* = 46), farmers (*n* = 13), and retired individuals (*n* = 284), all residing in Slovenia at the time of the survey. The full sample consisted of four subsamples. Respondents were recruited through a probability-based panel administered by the research agency Episcenter. The first subsample consisted of adults and was stratified by gender, age group, education level, and region to ensure national representativeness. The second subsample was representative of youth (15–29-year-olds), but was not included in the present study. The third subsample included the last-year students at three educational stages: primary, secondary school, and university. Finally, the fourth subsample included secondary school students from three educational tracks. To enable comparisons across educational stages and secondary tracks, last-year students were overrepresented in the full sample.

Table 1 shows the percentage distribution of adult individuals by demographics of the full sample, a weighted representative sample of adults, and official population statistics for adults [57]. As shown in Table 1, the weighted adult sample matched the population distribution. Last-year students were randomly selected from Slovenian primary and secondary schools and universities; however, due to overrepresentation, the school-enrolled youth and secondary-school student samples cannot be considered representative of educational stages or secondary schools.

### 3.2. Measurement

#### 3.2.1. Outcome Variables

Meat intake, our main outcome variable, was measured with three items. “Choose the answer that best describes your eating habits. I eat …” (1 = meat at most meals; 2 = meat at some meals, 3 = meat very rarely, 4 = no meat but fish, 5 = vegetarian, 6 = vegan).

Past meat reduction behaviour was measured with the following item: “Now we want to know whether the frequency of meat consumption (including red meat, processed meat, poultry, fish, and seafood) has changed for you personally over the last three years?” (1 = I have not eaten meat over a three-year period; 7 = I have increased the amount of meat in my diet considerably), which we recoded into a 6-point scale (1 = I have eliminated meat from my diet; 6 = I have increased the amount of meat in my diet considerably).

Intention to reduce meat consumption was measured with the item: “Please think about the period of the next month. Do you think that the frequency of meat consumption (including red meat, processed meat, poultry, fish, and seafood) will change for you personally over the next month?” (1 = I will continue to eat no meat; 7 = I will increase the amount of meat in my diet considerably), which we recoded into a 6-point scale (1 = I will eliminate meat from my diet; 6 = I will increase the amount of meat in my diet). Both past meat reduction and future intention to reduce meat were recoded from a 7-point into a 6-point scale by merging categories “I will continue to eat no meat”/“I have not eaten meat over a three-year period” with “I will maintain approximately the same amount of meat” response. This was conducted because both categories indicate no expected change in consumption level, which conceptually aligns them, and allowed us to ensure comparability across measures. We also reversed both of the variables, so higher values represent lower amounts of meat intake (i.e., individuals’ intention to reduce meat in the future, and that individuals have already reduced meat consumption in the past).

#### 3.2.2. Educational Predictor Variables

Among young people enrolled in education, we measured their current educational stage (1 = primary school; 2 = secondary school; 3 = tertiary school). Among secondary school students, we measured secondary educational track (1 = vocational; 2 = professional; 3 = general (i.e., gymnasium). Among adults, educational level was tapped with a question on education, with original values recoded into three categories (1 = primary school or less; 2 = secondary education; 3 = tertiary education).

#### 3.2.3. Climate Literacy

We focused on three main dimensions of climate literacy: knowledge, attitudes, and behaviour. The first dimension consisted of three subdimensions: climate science knowledge, knowledge of causes and consequences of climate change, and climate change mitigation.

To measure individuals’ climate science knowledge, as a first subdimension of the knowledge dimension of climate literacy, we used 7 items [58], all of which were questions with multiple choice, among which one of them was true, and the other ones were false. For instance: “What is the difference between weather and climate?” All of these items were recoded into 1 (correct answer) and 0 (incorrect answers). The rest of the items and the items for the other two subdimensions of climate knowledge (knowledge of causes and consequences of climate change and climate change mitigation) are presented in Table S1 in the Supplementary Materials. All three components of climate science knowledge were transformed to the same 1–5 scale before creating the final index. The causes and consequences and climate change mitigation indices (originally 1–4) were linearly rescaled to 1–5, and the factual knowledge items (originally coded 0–1 for incorrect/correct answers) were also rescaled to 1–5 linearly. These three standardised components were then averaged to form the overall climate science knowledge dimension, with higher scores indicating higher levels of climate knowledge.

Table S1 also presents items and scales for climate attitudes and behaviour, and their subdimensions. Specifically, climate attitudes consisted of (1) conviction and concern about climate change, (2) perceived ability to act to reduce global warming (self-efficacy), and (3) supporting government policies to reduce global warming.

The dimension of pro-environmental behaviour consisted of six subdimensions: energy saving, mobility, waste management, recycling, consumer behaviour, and indirect pro-environmental behaviour. Note that dietary habits were also included as items measuring pro-environmental behaviour, but we excluded them from the pro-environmental behaviour dimension in the present study, as our main outcome variable, meat intake, is also a pro-environmental behaviour.

All of the items used for climate attitudes and pro-environmental behaviour were recoded into a 5-point scale, and summation variables were created, in which higher levels represent higher levels of climate attitudes and pro-environmental behaviour.

### 3.3. Analytical Methods

We first present descriptive analyses of three climate literacy items for high school students, youth, and adults. To examine differences in mean scores between groups, we conducted one-way analyses of variance (ANOVA), followed by Tukey’s Honestly Significant Difference (HSD) post hoc test to identify which groups differed significantly from each other. We then calculated bivariate associations, followed by mediation analyses to examine if climate literacy mediates the relationship between educational predictors and three indicators of meat intake, separately for each sub-sample. For our analysis, we used SPSS software (version 30.0.0.0) and R (version 2025.05.0 + 496).

To ensure the validity of the mediation analyses, we checked various regression assumptions with “gvlma”, following Pena and Slate [59] and Kirshenbaum et al. [60]. We assessed these assumptions separately for each subsample. Because of the frequent deviations from normality and homoscedasticity, we used a robust maximum likelihood estimator (MLR) in mediation analyses to ensure robust and reliable parameter estimates [61]. Furthermore, assumption checks on the subsample of young people and adults revealed deviations from linearity. To satisfy this condition, we followed the approach from Krysko et al. [62], as we found that among the subsample of young individuals, linearity was not met on a path from education to past meat reduction via knowledge, on a path from education to meat intake via climate attitudes, and on a path from education to meat intake via pro-environmental behaviour. Therefore, we created a quadratic variable for knowledge in the first regression, log-transformed meat intake for the second regression, and created a quadratic variable for pro-environmental behaviour in the third regression. To avoid multicollinearity issues, we first centred the variables and then continued with their polynomial transformation.

**Figure 1:**
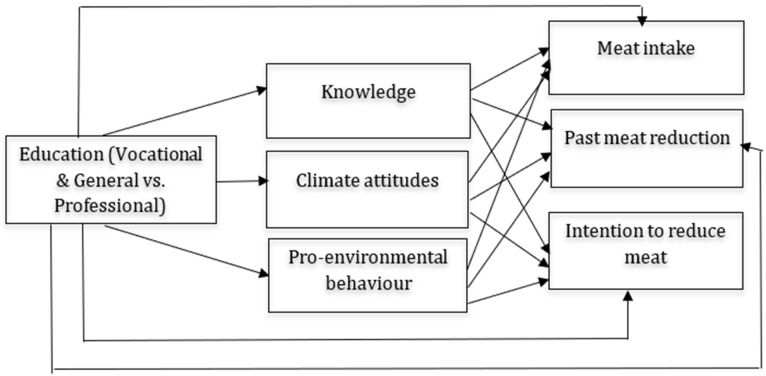
Mediation model for high-school students.

**Figure 2:**
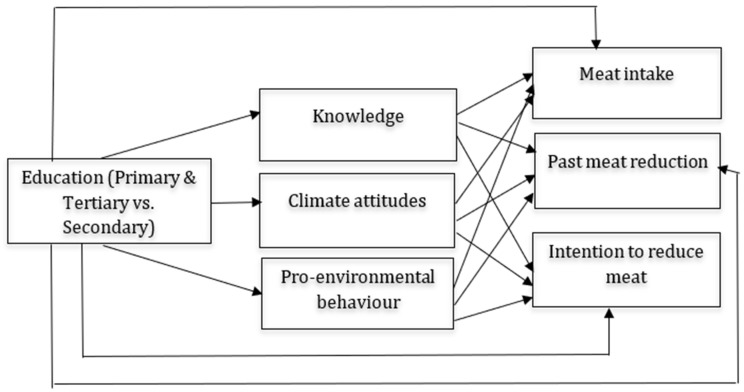
Mediation model for adults.

**Figure 3:**
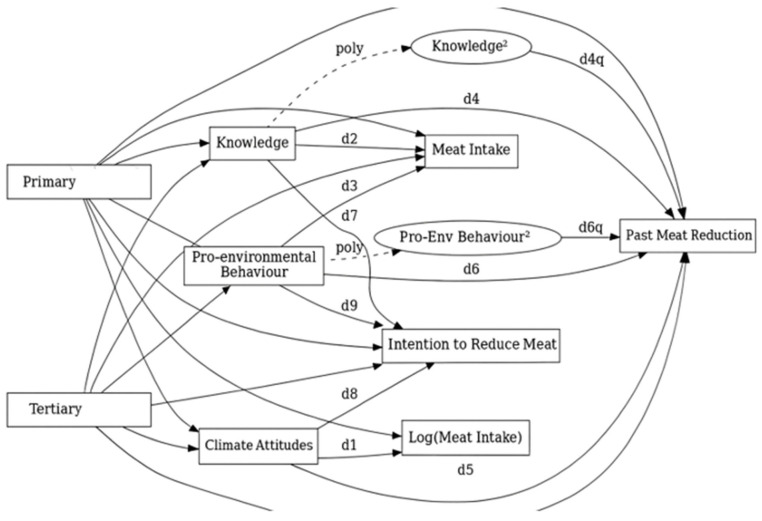
Mediation model for school-enrolled youth.

Linearity was also not satisfied in some regressions in the mediation model for adults, and polynomial transformation (log transformation, quadratic variable, cubic variable, etc.) did not help in mitigating this assumption. Because of that, we followed the approach from Trompeter et al. [63] and conducted bootstrapping analyses to provide bias-corrected *p*-values. All of the mediation models are presented in Figure 1, Figure 2 and Figure 3, whereas Figure 1 presents the mediation model for high-school students, Figure 2 presents the mediation model for adults, while Figure 3 presents the mediation model for school-enrolled youth. In each of the mediation analysis tables, we report unstandardized estimates, and the significance level was set at 0.05.

## 4. Results

### 4.1. Descriptive Statistics

First, we present results for descriptive statistics (mean, standard deviation) for three meat intake variables and dimensions and subdimensions of climate literacy for each of the three subsamples. Results are presented in Table 2. As for the first dimension of climate literacy, climate knowledge, adults chose the most correct answers (M = 3.41, SD = 0.55, *p* < 0.01), followed by high school students (M = 3.37, SD = 0.63) and school-enrolled youth (M = 3.27, SD = 0.65). Adults are also the ones with the highest correct answer score in two of the subdimensions: climate change causes and consequences (M = 3.79, SD = 0.71, *p* < 0.01) and climate change mitigation (M = 4.08, SD = 0.67, *p* < 0.05). The exception is the subdimension of climate science knowledge, where high school students show the highest level of knowledge (M = 2.62, SD = 0.93, *p* < 0.05).

As for the second dimension of climate literacy, climate attitudes, adults again showed more climate-favourable attitudes, in comparison to school-enrolled youth and high school students, in both the general attitudinal dimension (M = 3.27, SD = 0.7, *p* < 0.01) and its subdimensions (with exception of perceived ability to act to reduce global warming, as *p* > 0.05).

Findings on pro-environmental behaviour, the third dimension of climate literacy, similarly showed that adults reported the most pro-environmental behaviour (M = 3.59, SD = 0.46), and also among its subdimensions (with the exception of mobility subdimension), compared to school-enrolled youth and school students.

As for the meat consumption patterns, high school students, school-enrolled youth, and adults reported consuming meat at most meals or at least at some meals (M_(school-enrolled youth)_ = 1.67, SD_(school-enrolled youth)_ = 0.95; M_(high school students)_ = 1.55, SD_(high school students)_ = 0.87; M_(adults)_ = 2.08, SD_(adults)_ = 0.92), with adults consuming the lowest amount of meat (*p* < 0.01). As for the past meat reduction, the majority of adults somewhat reduced their meat intake in the past three years (M = 3.52, SD = 0.95), while the majority of school-enrolled youth and high school students ate the same amount of meat. Finally, the intention to reduce meat in the future was also highest among adults, although most of them planned to consume the same amount of meat in the future (M = 3.32, SD = 0.77, *p* < 0.01). Post hoc tests confirm that adults differ significantly from both youth groups, while school-enrolled youth and high school students do not differ from each other in each of the dimensions and sub-dimensions where results displayed significant differences among groups.

### 4.2. Bivariate Correlations

Table 3 presents results for Spearman correlations among the three educational characteristics (educational track for high school students, educational stage for school-enrolled youth, and educational level for adults), climate literacy dimensions, and three meat consumption variables. Among school-enrolled youth, higher levels of educational stage are associated with increased climate knowledge (ρ = 0.38, *p* < 0.05), climate attitudes (ρ = 0.13, *p* < 0.05), pro-environmental behaviour (ρ = 0.20, *p* < 0.05), with less meat intake (ρ = 0.10, *p* < 0.05) and increased past meat reduction (ρ = 0.09, *p* < 0.05). The correlations were similar among adults, with the addition that higher levels of education were also significantly associated with increased intention to reduce meat in the future (ρ = 0.09, *p* < 0.05). In all three of the subsamples, all three climate literacy dimensions were positively associated. Similarly, pro-environmental behaviour and higher levels of climate attitudes were consistently associated with lower meat intake and increased past and future meat reduction. Three meat variables were also positively associated across three subsamples.

### 4.3. Mediation Analyses

#### 4.3.1. Educational Stage Among School-Enrolled Youth

In the mediation analysis among school-enrolled youth, we examined educational stage as the predictor, with secondary educational stage being the reference category. Table 4 presents the results for the primary educational stage and Table 5 for the tertiary educational stage in comparison to the secondary educational stage. Table 4 indicates that in comparison to the secondary stage of education, those enrolled in primary education report significantly lower knowledge (β = −0.395, *p* < 0.01) and pro-environmental behaviour (β = −0.112, *p* < 0.01).

The direct effects of climate literacy dimensions showed mixed results. Specifically, increased pro-environmental behaviour is associated with higher meat intake (β = −0.043, *p* < 0.05). The results also showed that higher knowledge about climate change was associated with a lower intention to reduce meat consumption (β = −0.183, *p* < 0.01). On the other hand, an increase in climate attitudes is associated with lower meat intake (β = 0.007, *p* < 0.05). Similarly, increased climate attitudes are positively associated with intention to reduce meat in the future (β = 0.178, *p* < 0.01) and past meat reduction (β = 0.176, *p* < 0.01).

As for the indirect effects, only two of them are statistically significant. First, the primary educational stage is associated with lower meat intake, mainly through pro-environmental behaviour (β = 0.005, *p* < 0.05). And secondly, the primary educational stage has a positive impact on intention to reduce meat in the future, through higher levels of knowledge (β = 0.072, *p* < 0.01). Nevertheless, the total effects of the primary educational stage on all three outcome variables remained statistically insignificant, indicating that while primary education influences climate knowledge and pro-environmental behaviour, it does not have a direct impact on meat intake or meat reduction intentions overall. This suggests that education alone, without other factors, may not be sufficient to significantly change meat consumption patterns or intentions across the sample of school-enrolled youth.

Table 5 presents the impact of the tertiary educational stage of school-enrolled youth. In comparison to secondary educational stage, those in tertiary educational stage have increased climate knowledge (β = 0.159, *p* < 0.01), climate attitudes (β = 0.216, *p* < 0.01), pro-environmental behaviour (β = 0.115, *p* < 0.01), they consume less meat (β = 0.343, *p* < 0.01) and reduced it in the past three years (β = 0.317, *p* < 0.01). As for the indirect effects, meat intake was slightly lower among tertiary-educated school-enrolled youth through climate attitudes (β = 0.001, *p* < 0.05); however, meat intake was higher via pro-environmental behaviour (β = −0.005, *p* < 0.05).

Young people enrolled in the tertiary educational stage show more sustainable dietary patterns than their peers in secondary education, as they are more likely to have reduced their meat consumption in the past, mainly due to stronger climate attitudes (β = 0.038, *p* < 0.05). They also show significantly greater intention to reduce meat consumption in the future (β = 0.139, *p* < 0.05), and this effect is driven by two opposing indirect mechanisms: a positive effect via climate attitudes, and a negative effect via knowledge. Finally, they also consume less meat (β = 0.108, *p* < 0.01), with this effect partially explained by stronger climate attitudes, which positively mediate meat reduction, despite a small negative mediation via pro-environmental behaviour. Based on this, we confirm hypothesis H1(b), as a higher educational stage is associated with lower levels of meat consumption, past meat intake, and intention to reduce meat in the future. We also confirm the hypothesis H2, as results showed that climate literacy dimensions mediate the relationship between educational stage and meat consumption, with climate attitudes being the strongest mediator (RQ1).

#### 4.3.2. Educational Track Among High School Students

For high school students, we used the educational track as the predictor, and professional school as a reference category. Table 6 presents the results of mediation analyses for vocational school students, and Table 7 presents the results for general school students. In comparison to professional school students, vocational school students have lower levels of climate knowledge (β = −0.431, *p* < 0.01) and pro-environmental behaviour (β = −0.122, *p* < 0.05). However, vocational school students were more likely to report both past meat reduction (β = 0.429, *p* < 0.01) and intention to reduce meat (β = 0.339, *p* < 0.01). Direct effects showed that past meat reduction is associated with increased climate attitudes (β = 0.298, *p* < 0.01) and pro-environmental behaviour (β = 0.325, *p* < 0.05). Both climate literacy dimensions also predict intention to reduce meat in the future.

As for the indirect effects of vocational school on meat intake variables via knowledge, climate attitudes, and pro-environmental behaviour, none of them are statistically significant, indicating that vocational educational track does not have an effect on meat consumption through these mediators. However, the total effects on both past meat reduction (β = 0.386, *p* < 0.01) and intention to reduce meat (β = 0.347, *p* < 0.01) are statistically significant and positive, which indicates that the direct pathways are responsible for the significant and positive total effects.

Table 7 presents results for general school students in comparison to professional school students. In comparison to professional school students, students from general schools have higher knowledge (β = 0.275, *p* < 0.01), climate attitudes (β = 0.366, *p* < 0.01), and pro-environmental behaviour (β = 0.201, *p* < 0.01). They also report higher levels of past meat reduction (β = 0.288, *p* < 0.05).

As for the indirect effects, general school students are more likely to have reduced meat in the past, mainly through higher levels of climate attitudes (β = 0.106, *p* < 0.05), and have greater intention to reduce meat in the future, through both climate attitudes (β = 0.090, *p* < 0.05) and pro-environmental behaviour (β = 0.070, *p* < 0.05). Therefore, the statistical significance of the total effects of the general school track on past and future meat reduction is partly attributable to indirect pathways, particularly via climate attitudes and pro-environmental behaviour. Regarding hypothesis H2, climate literacy dimensions proved to be mediators between educational track and meat consumption, with climate attitudes being the strongest mediator (RQ1). As for the hypothesis H1(c), results are somewhat mixed, as the general school track predicted only past meat reduction, while the vocational educational track predicted both past and future meat reduction.

#### 4.3.3. Educational Level Among Adults

Finally, we present results from mediation analyses for adults in Table 8 and Table 9, with educational level as a predictor. Table 8 presents the results for adults with a primary educational level in comparison to adults with a secondary educational level. First, adults with a primary educational level report decreased climate attitudes (β = −0.284, *p* < 0.01) and pro-environmental behaviour (β = −0.347, *p* < 0.01). They are also less likely to have reduced meat in the past (β = −0.446, *p* < 0.01) and report a lower intention to reduce meat consumption in the future (β = −0.286, *p* < 0.01). Among adults, both climate attitudes and pro-environmental behaviour lower meat intake and increase past and future meat reduction, while higher levels of knowledge increase all three meat consumption outcomes.

Adults with a primary educational level show significantly less sustainable dietary behaviours compared to adults with higher levels of education, as they consume more meat (β = −0.298, *p* < 0.01), and this effect is strongly mediated by lower climate attitudes (β = −0.055, *p* < 0.01) and pro-environmental behaviour (β = −0.133, *p* < 0.01).

They are also less likely to have reduced their meat consumption in the past (β = −0.614, *p* < 0.01), and again, this is explained by lower climate attitudes (β = −0.057, *p* < 0.01) and pro-environmental behaviour (β = −0.122, *p* < 0.01).

Finally, they also show lower intention to reduce meat consumption (β = −0.420, *p* < 0.01), which is mediated by decreased climate attitudes (β = −0.051, *p* < 0.01) and lower pro-environmental behaviour (β = −0.090, *p* < 0.01).

Table 9 presents the results for adults with a tertiary educational level in comparison to those with a secondary educational level. First, we see that they have higher knowledge about climate change (β = 0.169, *p* < 0.01) and increased climate attitudes (β = 0.130, *p* < 0.05). They also consume less meat (β = 0.167, *p* < 0.01).

Mediation analysis showed that lower meat intake is mediated by lower knowledge (β = −0.064, *p* < 0.01), but also by increased climate attitudes (β = 0.025, *p* < 0.05). Climate knowledge also negatively mediates the relationship between adults with a tertiary educational level and past meat intake, but climate attitudes mediate this relationship positively. The same applies to the relationship between the tertiary educational level and intention to reduce meat. However, because of the statistical insignificance of these total effects, the conclusion is that a higher educational level does not predict past meat reduction or intention to reduce meat. However, a higher educational level among adults positively predicts lower meat intake, mainly through stronger climate attitudes. Based on this, we partly confirm hypothesis H1(a), as a higher educational level positively predicts only lower levels of meat intake. In this relationship, climate literacy was a significant mediator (H2), with climate attitudes being the strongest one (RQ1).

## 5. Discussion

In the present study, we examined whether climate literacy dimensions mediate the relationship between educational level, educational track, and educational stage and meat consumption patterns among adults, school-enrolled youth, and high school students from Slovenia. Previous studies have found that a higher educational level is associated with higher levels of pro-environmental behaviour and increased environmental concern [30,32,38]. Higher educational level is also associated with higher levels of sustainable dietary patterns, such as lower meat consumption [16,17]. But most of these studies mainly focused on one dimension of climate literacy (mostly on pro-environmental behaviour) and did not examine climate literacy as a multidimensional construct [33,34].

We found that individuals with higher educational level, track, and stage report lower levels of meat intake and stronger intentions to reduce meat. Among school-enrolled youth, the primary educational stage, compared to the secondary educational stage, had no statistically significant impact on meat consumption variables. On the other hand, the tertiary educational stage predicted lower levels of meat intake and a reduction in meat consumption in the past. We also found that vocational school students report more sustainable dietary patterns than their peers from the professional school track, as they were more likely to have already reduced their meat consumption in the past, and plan to do so in the future. Similarly, students from a general school also reduced their meat consumption in the past in comparison to their peers from the professional school track, but we found no statistically significant differences in their future intention to reduce meat intake. Previous studies rarely tested this relationship among youth. However, similar results were found in a study where youth with higher socioeconomic status (and with that higher education) reported a higher frequency of plant-based food consumption [64].

Finally, among adults, we found that primary educational level did not reduce their meat intake in the past, and they do not have the intention to reduce their levels in the future, compared to adults with a secondary educational level. Tertiary-educated adults, however, report lower meat intake compared to secondary-educated adults. The higher educated, however, are not significantly more likely to report past meat reduction and future intention. Conclusions regarding the subsample of adults are somewhat aligned with previous research, indicating that a higher educational level positively affects more sustainable dietary patterns (i.e., consuming less meat) [27,30,31].

We also tested which of the three dimensions of climate literacy (knowledge, attitudes, and behaviour) is the strongest mediator in the relationship between educational level, stage and track, and meat-related behaviours. Among school-enrolled youth, the tertiary educational stage, compared to the secondary educational stage, positively affects all three of the outcome variables, and among the three mediators, we found that climate attitudes are responsible for this positive effect. This was similar to high school students, where we found that the general school track positively affects both past meat reduction and intention to reduce meat compared to the professional school track, and the strongest and statistically significant mediator in these two relationships was climate attitudes. In addition, pro-environmental behaviour also mediates the relationship between general school track and intention to reduce meat.

Finally, among adults, primary educational level is associated with higher meat intake, low past meat reduction, and low future intention to reduce meat compared to secondary education, mainly because of lower pro-environmental behaviour and climate attitudes. Importantly, climate attitudes show a stronger association with meat intake patterns than pro-environmental behaviour in all three of the regressions. On the other hand, the tertiary educational level among adults decreased only meat intake. However, the reason behind this is unexpected, as adults with tertiary education have scored lower on knowledge of climate change. Still, despite knowing less about climate change, highly educated adults had more climate-friendly attitudes.

This contradicts past research, which finds that higher educational levels lead to better climate change knowledge [19,48,49]. In contrast to our results, previous studies found that higher levels of climate and environmental knowledge develop other dimensions of climate literacy and therefore enable individuals to act in an environmentally friendly way, as it is necessary for individuals to gain enough information about climate change that can motivate them to engage in pro-environmental behaviour [33,34,44]. But our results revealed that climate attitudes may have a higher impact than actual knowledge.

Therefore, we can somewhat confirm the claim that higher educational levels contribute to more sustainable dietary patterns indirectly through climate literacy, mainly through increased climate-friendly attitudes and partly through more frequent pro-environmental behaviour. Previous studies also confirm the relationship between educational attainment and climate literacy, as they found that individuals with higher educational levels have increased levels of environmental concern, which fosters their awareness of consequences for neglecting the environment, which in turn helps in developing more sustainable behaviours [1,18,35,36]. Moreover, Mata and colleagues [31] also explored the mediating role of environmental attitudes between education and processed meat consumption and found that higher-educated individuals have stronger environmental attitudes, and therefore consume lower levels of processed meat. Other studies similarly found that environmental attitudes positively predicted plant-based diet among college students [28] and that the attitude toward the impact of meat consumption on the environment predicts meat reduction [29].

Despite extending the literature on the importance of education, our study has several limitations. First, the study relies on cross-sectional data, which makes it difficult to establish causality between education, climate literacy, and meat consumption. Second, meat consumption and reduction, as well as the answers regarding climate literacy dimensions, were self-reported, which may be subject to social desirability bias. Third, although the study relies on a large and nationally representative sample from Slovenia (*n* = 2990), the findings are not directly generalizable to other cultural or socioeconomic contexts, as Slovenia is a high-income South-East European country, where meat consumption is frequent, and attitudes toward sustainable diets may differ elsewhere (e.g., in low-income countries with lower frequency of meat consumption) [27]. The findings may therefore not apply to other socioeconomic or cultural contexts. Moreover, while climate literacy was treated as a multidimensional concept (climate knowledge, attitudes, and behaviour), finer distinctions might yield deeper insights, such as measuring different types of climate knowledge or examining affective versus cognitive climate attitudes. Finally, the positive association between climate knowledge and meat intake contradicts prior literature, suggesting possible cultural specificity of Slovenia.

Based on our findings and study limitations, future research could develop longitudinal study designs to track changes in meat consumption as individuals progress through educational stages and levels to assess temporal and potentially causal effects. Future studies should also compare whether country-level characteristics moderate the role education and climate literacy play in meat intake patterns. Qualitative approaches should also be used, for example, semi-structured interviews and focus groups, to explore reasons vocational students show more sustainable meat-related behaviour than professional school students. Finally, future research should test whether integrating multidimensional climate literacy programmes into school curricula strengthens pro-environmental dietary behaviours.

Our findings suggest a few policy implications. First, with education system reforms, decision-makers could integrate climate literacy, especially climate-friendly attitudes, into school curricula at various educational levels, emphasising actionable steps like adopting sustainable diets (e.g., reducing meat consumption). Public awareness campaigns should target adults with lower educational levels by introducing the health and environmental benefits of reducing meat. Campaigns should also be based on attitude appeals, rather than just knowledge. There is also a possibility of implementing plant-based meals in schools and workplaces, particularly in regions with low educational attainment.

## 6. Conclusions

This study highlights that youth in the tertiary educational stage consume less meat, have reduced meat intake in the past, and have a future intention to reduce meat, with climate attitudes being the mediator in all three of the relationships. Moreover, we found that among adults, a tertiary educational level, compared to secondary, positively predicts a lower meat intake, mainly through increased climate attitudes. Adults with a primary educational level consume meat more frequently and show less willingness to reduce meat intake in the future, suggesting a need for targeted interventions. Additionally, both vocational and general secondary school students reported past and future meat reductions, compared to professional school students. The findings underscore that climate literacy, especially climate attitudes, plays a key role in shaping sustainable dietary patterns, and that climate knowledge alone may be insufficient. Policymakers should prioritise attitude-based education, school programmes, and accessible campaigns to promote meat reduction among individuals with lower educational backgrounds. Future research should explore cultural and socioeconomic factors influencing dietary choices regarding meat consumption, including the mediating role of climate literacy dimensions, to refine strategies for broader impact, such as designing targeted climate education programmes and sustainable food policies.

## Figures and Tables

**Table 1:** Descriptive statistics.

		Full Sample	Representative Sample of Adults (Weighted)	Population of Slovenia
		**%**	**%**	**%**
Gender	Male	45.8%	50.0%	50.0%
	Female	54.2%	50.0%	50.0%
Status	Active (employed part-time, full-time, etc.)	25.4%	47.2%	55.1%
	Retired	9.5%	32.9%	29.6%
	Other (students)	65.0%	19.9%	15.3%
Education	Primary or less	2.1%	18.7%	19.0%
	Secondary	58.1%	54.1%	54.0%
	Tertiary	39.5%	27.2%	27.0%
Age	18–34 years old	26.2%	21.5%	22.0%
	35–44 years old	13.4%	17.3%	17.0%
	45–54 years old	19.7%	17.6%	18.0%
	55–64 years old	19.4%	17.0%	17.0%
	older than 65 years	21.3%	26.5%	26.0%

Source: [57].

**Table 2:** Descriptive statistics: meat intake, past meat reduction, intention to reduce meat, dimensions, and subdimensions of climate literacy.

		School-Enrolled Youth		High School Students		Adults		
		**M**	**SD**	**M**	**SD**	**M**	**SD**	**Groups**
Dimensions	Knowledge **	3.27	0.65	3.37	0.63	3.41	0.55	a b a
	Climate attitudes **	3.1	0.6	3.04	0.63	3.27	0.7	b b a
	Pro-environmental behaviour **	3.24	0.41	3.25	0.44	3.59	0.46	b b a
Sub-dimensions of knowledge	Climate science knowledge *	2.46	0.99	2.62	0.93	2.52	0.86	ab b a
	Causes and consequences of climate change **	3.66	0.6	3.71	0.65	3.79	0.71	ab b a
	Climate change mitigation *	4	0.63	4.02	0.65	4.08	0.67	ab b a
Sub-dimensions of climate attitudes	Conviction and concern about the climate change **	3.14	0.68	3.12	0.71	3.28	0.76	b b a
	Perceived ability to act to reduce global warming (self-efficacy)	3.46	0.73	3.45	0.76	3.5	0.8	a a a
	Supporting government policies to reduce global warming **	2.7	1.01	2.53	1.04	3.04	1.07	b c a
Sub-dimensions of pro-environmental behaviour	Energy saving **	3.46	0.66	3.5	0.67	3.86	0.63	b b a
	Mobility	3.6	0.73	3.58	0.75	3.59	0.71	a a a
	Waste management **	3.09	0.52	3.09	0.57	3.36	0.58	b b a
	Recycling **	3.44	0.68	3.45	0.7	4.03	0.71	b b a
	Consumer behaviour **	3.02	0.49	3.01	0.52	3.47	0.59	b b a
	Indirect pro-environmental behaviour **	2.85	0.55	2.88	0.57	3.21	0.57	b b a
Meat consumption and reduction	Meat eating **	1.67	0.95	1.55	0.87	2.08	0.92	b b a
	Past meat reduction **	3.05	1.1	2.92	1.08	3.52	0.95	b b a
	Intention to reduce meat **	3.09	0.89	3.04	0.91	3.32	0.77	b b a

Note. Asterisks next to variable names represent statistically significant differences between subsamples (* *p* < 0.05, ** *p* < 0.01). Letters in the “Groups” column indicate results of post-hoc pairwise comparisons using Tukey’s HSD. Means sharing the same letter are not significantly different from each other at *p* < 0.05.

**Table 3:** Spearman correlations among variables for young, high school students and adults.

School-Enrolled Youth							
		1	2	3	4	5	6
	1. Educational stage						
	2. Knowledge	0.38 *					
	3. Climate attitudes	0.13 *	0.35 *				
	4. Pro-environmental behaviour	0.20 *	0.39 *	0.43*			
	5. Meat intake	0.10 *	−0.01	0.17 *	0.14 *		
	6. Past meat reduction	0.09 *	−0.03	0.17 *	0.11 *	0.45 *	
	7. Intention to reduce meat	0.04	−0.04	0.11 *	0.07 *	0.30 *	0.44 *
High school students							
		1	2	3	4	5	6
	1. Educational track						
	2. Knowledge	0.44 *					
	3. Climate attitudes	0.28 *	0.31 *				
	4. Pro-environmental behaviour	0.28 *	0.33 *	0.48 *			
	5. Meat intake	0.06	0.03	0.19 *	0.17 *		
	6. Past meat reduction	0.06	0.09	0.23 *	0.21 *	0.41 *	
	7. Intention to reduce meat	−0.01	−0.01	0.19 *	0.21 *	0.30 *	0.47 *
Adults							
		1	2	3	4	5	6
	1. Educational level						
	2. Knowledge	0.13 *					
	3. Climate attitudes	0.20 *	0.48 *				
	4. Pro-environmental behaviour	0.27 *	0.21 *	0.42 *			
	5. Meat intake	0.20 *	−0.04 *	0.19 *	0.30 *		
	6. Past meat reduction	0.16 *	−0.01	0.17 *	0.23 *	0.48 *	
	7. Intention to reduce meat	0.09 *	0.01	0.19 *	0.19 *	0.28 *	0.51 *

Note. * *p* < 0.05.

**Table 4:** Mediation role of climate literacy dimensions in the relationship between education and meat intake, past meat reduction, and intention to reduce meat for school-enrolled youth in the primary educational stage.

Paths		Estimate	SE	95% CI [LL, UL]
	Primary educational stage → Knowledge (a1)	−0.395 **	0.043	[−0.480, −0.311]
	Primary educational stage → Climate attitudes (a3)	0.015	0.041	[−0.066, 0.096]
	Primary educational stage → Pro-environmental behaviour (a5)	−0.112 **	0.03	[−0.171, −0.052]
	Climate attitudes → Meat intake (log) (d1)	0.007 *	0.003	[0.000, 0.013]
	Knowledge → Meat intake (d2)	0.004	0.011	[−0.018, 0.026]
	Pro-environmental behaviour → Meat intake (d3)	−0.043 *	0.017	[−0.076, −0.010]
	Knowledge→ Past meat reduction (d4)	−0.056	0.064	[−0.180, 0.069]
	Knowledge (squared) → Past meat reduction (d4q)	0.06	0.044	[−0.027, 0.147]
	Climate attitudes → Past meat reduction (d5)	0.176 **	0.058	[0.062, 0.290]
	Pro-environmental behaviour → Past meat reduction (d6)	0.048	0.079	[−0.107, 0.203]
	Pro-environmental behaviour (squared) → Past meat reduction (d6q)	−0.100	0.106	[−0.308, 0.107]
	Knowledge → Intention to reduce meat (d7)	−0.183 **	0.057	[−0.294, −0.072]
	Climate attitudes → Intention to reduce meat (d8)	0.178 **	0.052	[0.076, 0.280]
	Pro-environmental behaviour → Intention to reduce meat (d9)	0.124	0.072	[−0.018, 0.266]
	Primary educational stage → Meat intake (log) (c1)	0.029	0.020	[−0.010, 0.069]
	Primary educational stage → Meat intake (c3)	0.083	0.064	[−0.042, 0.207]
	Primary educational stage → Past meat reduction (c5)	0.053	0.081	[−0.106, 0.212]
	Primary educational stage → Intention to reduce meat (c7)	−0.009	0.069	[−0.144, 0.126]
Indirect effects	On Meat intake via Climate attitudes	0.000	0.000	[−0.000, 0.001]
	On Meat intake via Knowledge	−0.002	0.004	[−0.010, 0.007]
	On Meat intake via Pro-environmental behaviour	0.005 *	0.002	[0.000, 0.009]
	On Past meat reduction via Knowledge (poly)	−0.002	0.035	[−0.071, 0.068]
	On Past meat reduction via Climate attitudes	0.003	0.007	[−0.012, 0.017]
	On Past meat reduction via Pro-environmental behaviour (poly)	0.006	0.016	[−0.026, 0.038]
	On Intention to reduce meat via Knowledge	0.072 **	0.024	[0.026, 0.119]
	On Intention to reduce meat via Climate attitudes	0.003	0.007	[−0.012, 0.017]
	On Intention to reduce meat via Pro-environmental behaviour	−0.014	0.009	[−0.031, 0.004]
Total effects	Meat intake	0.033	0.021	[−0.008, 0.010]
	Past meat reduction	0.060	0.082	[−0.101, 0.220]
	Intention to reduce meat	0.052	0.067	[−0.079, 0.183]

Note. * *p* < 0.05, ** *p* < 0.01.

**Table 5:** Mediation role of climate literacy dimensions in the relationship between education and meat intake, past meat reduction, and intention to reduce meat for school-enrolled youth in tertiary educational stage.

Paths		Estimate	SE	95% CI [LL, UL]
	Tertiary educational stage → Knowledge (a2)	0.159 **	0.047	[0.067, 0.252]
	Tertiary educational stage → Climate attitudes (a4)	0.216 **	0.047	[0.124, 0.309]
	Tertiary educational stage → Pro-environmental behaviour (a6)	0.115 **	0.029	[0.057, 0.172]
	Climate attitudes → Meat intake (log) (d1)	0.007 *	0.003	[0.000, 0.013]
	Knowledge → Meat intake (d2)	0.004	0.011	[−0.018, 0.026]
	Pro-environmental behaviour → Meat intake (d3)	−0.043 *	0.017	[−0.076, −0.010]
	Knowledge → Past meat reduction (d4)	−0.056	0.064	[−0.180, 0.069]
	Knowledge (squared) → Past meat reduction (d4q)	0.06	0.044	[−0.027, 0.147]
	Climate attitudes → Past meat reduction (d5)	0.176 **	0.058	[0.062, 0.290]
	Pro-environmental behaviour → Past meat reduction (d6)	0.048	0.079	[−0.107, 0.203]
	Pro-environmental behaviour (squared) → Past meat reduction (d6q)	−0.100	0.106	[−0.308, 0.107]
	Knowledge → Intention to reduce meat (d7)	−0.183 **	0.057	[−0.294, −0.072]
	Climate attitudes → Intention to reduce meat (d8)	0.178 **	0.052	[0.076, 0.280]
	Pro-environmental behaviour → Intention to reduce meat (d9)	0.124	0.072	[−0.018, 0.266]
	Tertiary educational stage → Meat intake (log) (c2)	0.111 **	0.023	[0.066, 0.157]
	Tertiary educational stage → Meat intake (c4)	0.343 **	0.075	[0.197, 0.490]
	Tertiary educational stage → Past meat reduction (c6)	0.317 **	0.08	[0.159, 0.474]
	Tertiary educational stage → Intention to reduce meat (c8)	0.116	0.059	[−0.001, 0.232]
Indirect effects	On Meat intake via Climate attitudes	0.001 **	0.001	[0.000, 0.003]
	On Meat intake via Knowledge	0.001	0.002	[−0.003, 0.004]
	On Meat intake via Pro-environmental behaviour	−0.005 *	0.002	[−0.010, −0.000]
	On Past meat reduction via Knowledge (poly)	0.001	0.014	[−0.027, 0.029]
	On Past meat reduction via Climate attitudes	0.038 *	0.015	[0.009, 0.068]
	On Past meat reduction via Pro-environmental behaviour (poly)	−0.006	0.017	[−0.039, 0.027]
	On Intention to reduce meat via Knowledge	−0.029 *	0.012	[−0.053, −0.006]
	On Intention to reduce meat via Climate attitudes	0.039 **	0.014	[0.010, 0.067]
	On Intention to reduce meat via Pro-environmental behaviour	0.014	0.009	[−0.004, 0.032]
Total effects	Meat intake	0.108 **	0.023	[0.062, 0.154]
	Past meat reduction	0.350 **	0.081	[0.191, 0.509]
	Intention to reduce meat	0.139 *	0.061	[0.019, 0.259]

Note. * *p* < 0.05, ** *p* < 0.01.

**Table 6:** Mediation role of climate literacy dimensions in the relationship between education and meat intake, past meat reduction, and intention to reduce meat for vocational high school students.

Paths		Estimate	SE	95% CI [LL, UL]
	Vocational school → Knowledge (a1)	−0.431 **	0.076	[−0.580, −0.282]
	Vocational school → Climate attitudes (a3)	−0.080	0.072	[−0.221, 0.061]
	Vocational school → Pro-environmental behaviour (a5)	−0.122 *	0.048	[−0.216, −0.028]
	Knowledge → Meat intake (b1)	−0.051	0.086	[−0.219, 0.117]
	Climate attitudes → Meat intake (b2)	0.112	0.071	[−0.027, 0.251]
	Pro-environmental behaviour → Meat intake (b3)	0.143	0.130	[−0.112, 0.398]
	Knowledge → Past meat reduction (b4)	−0.045	0.112	[−0.265, 0.175]
	Climate attitudes → Past meat reduction (b5)	0.290 **	0.104	[0.086, 0.494]
	Pro-environmental behaviour → Past meat reduction (b6)	0.325 *	0.147	[0.037, 0.613]
	Knowledge → Intention to reduce meat (b7)	−0.161	0.083	[−0.324, 0.002]
	Climate attitudes → Intention to reduce meat (b8)	0.246 **	0.093	[0.064, 0.428]
	Pro-environmental behaviour → Intention to reduce meat (b9)	0.348 **	0.122	[0.109, 0.587]
	Vocational school → Meat intake (c1)	0.145	0.114	[−0.078, 0.368]
	Vocational school → Past meat reduction (c3)	0.429 **	0.139	[0.156, 0.702]
	Vocational school → Intention to reduce meat (c5)	0.339 **	0.124	[0.096, 0.582]
Indirect effects	On Meat intake via Knowledge	0.022	0.037	[−0.051, 0.095]
	On Past meat reduction via Knowledge	0.020	0.049	[−0.076, 0.116]
	On Intention to reduce meat via Knowledge	0.069	0.038	[−0.005, 0.143]
	On Meat intake via Climate attitudes	−0.009	0.010	[−0.029, 0.011]
	On Past meat reduction via Climate attitudes	−0.023	0.022	[−0.066, 0.020]
	On intention to reduce meat via Climate attitudes	−0.020	0.019	[−0.057, 0.017]
	On Meat intake via Pro-environmental behaviour	−0.017	0.017	[−0.050, 0.016]
	On Past meat reduction via Pro-environmental behaviour	−0.040	0.023	[−0.085, 0.005]
	On Intention to reduce meat via Pro-environmental behaviour	−0.043	0.023	[−0.088, 0.002]
Total effects	Meat intake	0.141	0.113	[−0.080, 0.362]
	Past meat reduction	0.386 **	0.142	[0.108, 0.664]
	Intention to reduce meat	0.347 **	0.127	[0.098, 0.596]

Note. * *p* < 0.05, ** *p* < 0.01.

**Table 7:** Mediation role of climate literacy dimensions in the relationship between education and meat intake, past meat reduction, and intention to reduce meat for general high school students.

Paths		Estimate	SE	95% CI [LL, UL]
	General school → Knowledge (a2)	0.275 **	0.06	[0.157, 0.393]
	General school → Climate attitudes (a4)	0.366 **	0.072	[0.225, 0.507]
	General school → Pro-environmental behaviour (a6)	0.201 **	0.052	[0.099, 0.303]
	Knowledge → Meat intake (b1)	−0.051	0.086	[−0.219, 0.117]
	Climate attitudes → Meat intake (b2)	0.112	0.071	[−0.027, 0.251]
	Pro-environmental behaviour → Meat intake (b3)	0.143	0.13	[−0.112, 0.398]
	Knowledge → Past meat reduction (b4)	−0.045	0.112	[−0.265, 0.175]
	Climate attitudes → Past meat reduction (b5)	0.290 **	0.104	[0.086, 0.494]
	Pro-environmental behaviour → Past meat reduction (b6)	0.325 *	0.147	[0.037, 0.613]
	Knowledge → Intention to reduce meat (b7)	−0.161	0.083	[−0.324, 0.002]
	Climate attitudes → Intention to reduce meat (b8)	0.246 **	0.093	[0.064, 0.428]
	Pro-environmental behaviour → Intention to reduce meat (b9)	0.348 **	0.122	[0.109, 0.587]
	General school → Meat intake (c2)	0.125	0.104	[−0.079, 0.329]
	General school → Past meat reduction (c4)	0.288 *	0.129	[0.035, 0.541]
	General school → Intention to reduce meat (c6)	0.129	0.099	[−0.065, 0.323]
Indirect effects				
	On Meat intake via Knowledge	−0.014	0.024	[−0.061, 0.033]
	On Past meat reduction via Knowledge	−0.012	0.031	[−0.073, 0.049]
	On Intention to reduce meat via Knowledge	−0.044	0.025	[−0.093, 0.005]
	On Meat intake via Climate attitudes	0.041	0.027	[−0.012, 0.094]
	On Past meat reduction via Climate attitudes	0.106 *	0.044	[0.020, 0.192]
	On intention to reduce meat via Climate attitudes	0.090 *	0.039	[0.014, 0.166]
	On Meat intake via Pro-environmental behaviour	0.029	0.028	[−0.026, 0.084]
	On Past meat reduction via Pro-environmental behaviour	0.065	0.036	[−0.005, 0.135]
	On Intention to reduce meat via Pro-environmental behaviour	0.070 *	0.03	[0.011, 0.129]
Total effects	Meat intake	0.181	0.096	[−0.007, 0.369]
	Past meat reduction	0.447 ***	0.116	[0.220, 0.674]
	Intention to reduce meat	0.245 **	0.09	[0.068, 0.422]

Note. * *p* < 0.05, ** *p* < 0.01, *** *p* < 0.001.

**Table 8:** Mediation role of climate literacy dimensions in the relationship between education and meat intake, past meat reduction, and intention to reduce meat for adults with a primary educational level.

Paths		Estimate	SE	95% CI [LL, UL]
	Primary educational level → Knowledge (a1)	−0.047	0.056	[−0.156, 0.062]
	Primary educational level → Climate attitudes (a3)	−0.284 **	0.06	[−0.401, −0.167]
	Primary educational level → Pro-environmental behaviour (a5)	−0.347 **	0.041	[−0.428, −0.266]
	Knowledge → Meat intake (b1)	−0.378 **	0.082	[−0.540, −0.217]
	Climate attitudes → Meat intake (b2)	0.195 **	0.057	[0.084, 0.306]
	Pro-environmental behaviour → Meat intake (b3)	0.384 **	0.079	[0.230, 0.538]
	Knowledge → Past meat reduction (b4)	−0.248 **	0.073	[−0.391, −0.104]
	Climate attitudes → Past meat reduction (b5)	0.201 **	0.054	[0.096, 0.307]
	Pro-environmental behaviour → Past meat reduction (b6)	0.353 **	0.078	[0.201, 0.505]
	Knowledge → Intention to reduce meat (b7)	−0.124 *	0.056	[−0.233, −0.015]
	Climate attitudes → Intention to reduce meat (b8)	0.178 **	0.045	[0.089, 0.267]
	Pro-environmental behaviour → Intention to reduce meat (b9)	0.258 **	0.064	[0.133, 0.383]
	Primary educational level → Meat intake (c1)	−0.127	0.083	[−0.290, 0.035]
	Primary educational level → Past meat reduction (c3)	−0.446 **	0.097	[−0.636, −0.256]
	Primary educational level → Intention to reduce meat (c5)	−0.286 **	0.079	[−0.441, −0.130]
Indirect effects	On Meat intake via Knowledge	0.018	0.022	[−0.026, 0.061]
	On Past meat reduction via Knowledge	0.012	0.015	[−0.017, 0.041]
	On Intention to reduce meat via Knowledge	0.006	0.007	[−0.008, 0.020]
	On Meat intake via Climate attitudes	−0.055 **	0.02	[−0.095, −0.016]
	On Past meat reduction via Climate attitudes	−0.057 **	0.02	[−0.096, −0.019]
	On intention to reduce meat via Climate attitudes	−0.051 **	0.017	[−0.084, −0.017]
	On Meat intake via Pro-environmental behaviour	−0.133 **	0.029	[−0.191, −0.076]
	On Past meat reduction via Pro-environmental behaviour	−0.122 **	0.029	[−0.180, −0.065]
	On Intention to reduce meat via Pro-environmental behaviour	−0.090 **	0.025	[−0.138, −0.041]
Total effects	Meat intake	−0.298 **	0.088	[−0.470, −0.127]
	Past meat reduction	−0.614 **	0.101	[−0.813, −0.415]
	Intention to reduce meat	−0.420 **	0.085	[−0.586, −0.254]

Note. * *p* < 0.05, ** *p* < 0.01.

**Table 9:** Mediation role of climate literacy dimensions in the relationship between education and meat intake, past meat reduction, and intention to reduce meat for adults with tertiary educational level.

Paths		Estimate	SE	95% CI [LL, UL]
	Tertiary educational level → Knowledge (a2)	0.169 **	0.039	[0.093, 0.245]
	Tertiary educational level → Climate attitudes (a4)	0.130 *	0.056	[0.021, 0.239]
	Tertiary educational level → Pro-environmental behaviour (a6)	0.04	0.033	[−0.024, 0.104]
	Knowledge → Meat intake (b1)	−0.378 **	0.082	[−0.540, −0.217]
	Climate attitudes → Meat intake (b2)	0.195 **	0.057	[0.084, 0.306]
	Pro-environmental behaviour → Meat intake (b3)	0.384 **	0.079	[0.230, 0.538]
	Knowledge → Past meat reduction (b4)	−0.248 **	0.073	[−0.391, −0.104]
	Climate attitudes → Past meat reduction (b5)	0.201 **	0.054	[0.096, 0.307]
	Pro-environmental behaviour → Past meat reduction (b6)	0.353 **	0.078	[0.201, 0.505]
	Knowledge → Intention to reduce meat (b7)	−0.124 *	0.056	[−0.233, −0.015]
	Climate attitudes → Intention to reduce meat (b8)	0.178 **	0.045	[0.089, 0.267]
	Pro-environmental behaviour → Intention to reduce meat (b9)	0.258 **	0.064	[0.133, 0.383]
	Tertiary educational level → Meat intake (c2)	0.167 **	0.061	[0.048, 0.286]
	Tertiary educational level → Past meat reduction (c4)	−0.041	0.059	[−0.157, 0.075]
	Tertiary educational level → Intention to reduce meat (c6)	−0.094	0.051	[−0.194, 0.006]
Indirect effects	On Meat intake via Knowledge	−0.064 **	0.02	[−0.103, −0.025]
	On Past meat reduction via Knowledge	−0.042 **	0.015	[−0.072, −0.012]
	On Intention to reduce meat via Knowledge	−0.021 *	0.011	[−0.042, −0.000]
	On Meat intake via Climate attitudes	0.025 *	0.013	[0.001, 0.050]
	On Past meat reduction via Climate attitudes	0.026 *	0.013	[−0.000, 0.052]
	On intention to reduce meat via Climate attitudes	0.023 *	0.012	[0.000, 0.046]
	On Meat intake via Pro-environmental behaviour	0.015	0.013	[−0.010, 0.041]
	On Past meat reduction via Pro-environmental behaviour	0.014	0.012	[−0.009, 0.038]
	On Intention to reduce meat via Pro-environmental behaviour	0.01	0.009	[−0.007, 0.028]
Total effects	Meat intake	0.144 *	0.063	[0.020, 0.267]
	Past meat reduction	−0.043	0.06	[−0.161, 0.075]
	Intention to reduce meat	−0.082	0.052	[−0.183, 0.019]

Note. * *p* < 0.05, ** *p* < 0.01.

## Data Availability

The original contributions presented in the study are included in the article, further inquiries can be directed to the corresponding author.

## Supplementary Materials

The following supporting information can be downloaded at: https://www.mdpi.com/article/10.3390/foods14193333/s1, Table S1: Measurement instrument: dimensions and subdimensions of climate literacy.
