# Supplementary material for: Education and Meat Consumption and Reduction: The Mediating Role of Climate Literacy
Source: Foods. 2025 Sep 25;14(19):3333. doi: 10.3390/foods14193333 (PMC12523753; doi:10.3390/foods14193333)
Supplement: Supplementary file 1 [file foods-14-03333-s001.zip › foods-3877487-supplementary.pdf]

Table S1. Measurement instrument: dimensions and subdimensions of climate literacy

Dimension	Subdimension	Item	Scale
**Knowledge**	**Climate science knowledge**	What is the difference between weather and climate?	**A. Weather is a day-to-day event while climate is a consistent pattern over a year or longer.** B. Weather is local, but climate is global. C. Weather is predicable but climate is not. D. Weather includes more variables like moisture and wind while climate just focuses on temperature and precipitation. E. I don't know.
		Which greenhouse gas are we most concerned about if we want to reduce emissions to mitigate global warming?	**A. carbon dioxide (CO2)**<br>B. water vapor (H2O)<br>C. methane (CH4)<br>D. oxygen (O2)<br>E. ozone (O3)
		Which of the following best describes how greenhouse gases can affect global warming?	A. They can destroy certain gases in the atmosphere. B. They bend and magnify sunlight entering the atmosphere. C. They can trap certain chemicals in the atmosphere. D. They absorb moisture from the atmosphere. E. **They absorb energy at certain wavelengths but not all.**
		What is the relationship between the greenhouse effect and global warming?	A. The greenhouse effect and global climate change are the same thing.<br>**B. An increase in the greenhouse effect may be causing global climate change.**<br>C. Global climate change may be causing an increase in the greenhouse effect.<br>D. The greenhouse effect and global climate change are likely unrelated.<br>E. There is no definite proof that either the greenhouse effect or global climate change exist.

	What is the main characteristic of greenhouse gases?	A. They can destroy certain molecules in the atmosphere. B. They bend and magnify sunlight entering the atmosphere. C. They can trap certain molecules in the atmosphere. D. They absorb moisture from the atmosphere. E. **They absorb energy at certain wavelengths but not all.**
	The Earth's atmosphere is warmer than it would be without the greenhouse effect. Which wavelength of energy absorbed by the atmosphere is mainly responsible for the temperature rise?	A. radio **B. infrared** C. visible D. ultraviolet E. x-ray
	Would the greenhouse effect have existed if human civilisation had never come into existence?	**A. Yes, the greenhouse effect is caused by naturally occurring gases in the atmosphere**. B. Yes, the greenhouse effect is caused by plants giving off gases during photosynthesis. C. No, the greenhouse effect is caused by humans burning fossil fuels and releasing pollutants. D. No, the greenhouse effect is caused by humans depleting ozone in the atmosphere. E. No, there is no conclusive evidence that the greenhouse effect exists.
**Causes and consequences of climate change**	Global warming is happening because too many of the sun’s rays get to the earth. (R)	1 = not true; 4 = certainly true
	Melting of snow and ice from land areas will likely result in rising sea levels.	1 = not true; 4 = certainly true
	Global climate change is accelerated by the melting of snow and ice covered surfaces.	1 = not true; 4 = certainly true

		Please indicate to the best of your knowledge which of the following is a cause of global climate change. Use the following answer choices:	Burning fossil fuels	1 = definitely not; 4 = definitely
			Livestock (beef and pork) production	1 = definitely not; 4 = definitely
			The ozone hole in the upper atmosphere.	1 = definitely not; 4 = definitely
	**Climate change mitigation**	Which of the following actions will help reduce or slow down climate change? Select from the following answer choices:	Building more nuclear power stations instead of coal power stations.	1 = it certainly doesn't help; 4 = it certainly helps
			Planting more trees in the world.	1 = it certainly doesn't help; 4 = it certainly helps
			Making more of our electricity from renewable energy resources.	1 = it certainly doesn't help; 4 = it certainly helps
			Recycling more.	1 = it certainly doesn't help; 4 = it certainly helps
			Not wasting electricity.	1 = it certainly doesn't help; 4 = it certainly helps
			Reducing air pollution from toxic chemicals.	1 = it certainly doesn't help; 4 = it certainly helps
**Climate attitudes**	**Conviction and concern about the climate change**		How convinced are you that global warming is happening?	1 = I'm not sure at all/; 5 = I'm pretty sure
			When do you think global warming will start to have serious impacts on people around the world?	1 = Never; 5 = It's already having serious impacts
		Please indicate how serious you consider global warming to be a threat to each of the following, using the following answer choices:	Plants and animals	1 = not a threat; 5 = a threat of extreme urgency
			People in Slovenia	1 = not a threat; 5 = a threat of extreme urgency

			Your local community	1 = not a threat; 5 = a threat of extreme urgency
			People in other countries	1 = not a threat; 5 = a threat of extreme urgency
			Overall, how concerned are you about climate change?	1 = I'm not worried at all; 5 = very worried
	**Perceived ability to act to reduce global warming (self-efficacy)**		I believe I can take actions that will help reduce global warming.	1 = strongly disagree; 5 = strongly agree
			I believe that, by working with others, we can take actions that will help reduce global warming.	1 = strongly disagree; 5 = strongly agree
			The actions of a single person won't make any difference in reducing global warming. (R)	1 = strongly disagree; 5 = strongly agree
			The actions of a single country like the United States won't make any difference in reducing global warming. (R)	1 = strongly disagree; 5 = strongly agree
	**Supporting government policies to reduce global warming**	How do you feel about each of the following possible ways for the federal or state governments to reduce global warming? Use the following answer choices:	Requiring automakers to increase the fuel efficiency of cars, trucks, and SUVs to 35 miles per gallon, even if it meant a new car would cost up to $500 more to buy.	1 = strongly oppose; 5 = strongly favor
			Increasing taxes on gasoline so people either drive less or buy cars that use less gas.	1 = strongly oppose; 5 = strongly favor
**Pro-environmental behaviour**	**Energy saving**		After one day of use, my sweaters or trousers go into the laundry. (R)	1 = almost never or never; 5 = almost always or always
			In turn of the light when I leave the room.	1 = almost never or never; 5 = almost always or always

	After use, I turn off my computer.	1 = almost never or never; 5 = almost always or always
	In the winter, I turn down the heat when I leave my room for more than 4 hours.	1 = almost never or never; 5 = almost always or always
	In the winter, it is warm enough in my room to only wear a T-shirt. (R)	1 = almost never or never; 5 = almost always or always
	In hotels, I have the towels changed daily./ In hotels, me and my family change towels daily. (R)	1 = almost never or never; 5 = almost always or always
**Mobility**	I'm being driven around in a car./ I drive a car. (R)	1 = almost never or never; 5 = almost always or always
	I cycle to school/work, use public transport or walk.	1 = almost never or never; 5 = almost always or always
	For short distances, I walk or cycle instead of using a car or asking for transport.	1 = almost never or never; 5 = almost always or always
**Waste management**	I buy canned drinks or have them bought for me by people close to me. (R)	1 = almost never or never; 5 = almost always or always
	I buy drinks in returnable bottles or have them bought for me by people close to me.	1 = almost never or never; 5 = almost always or always
	I buy products in refillable containers or have them bought for me by people close to me.	1 = almost never or never; 5 = almost always or always
	If I am offered a plastic bag in a shop, I take it or buy it. (R)	1 = almost never or never; 5 = almost always or always
	I take disposable drinks with me on trips (e.g. Coca-Cola, juice in a tetrapack, etc). (R)	1 = almost never or never; 5 = almost always or always

	At my parties and get-togethers with friends, we use plastic cutlery and paper cups. (R)	1 = almost never or never; 5 = almost always or always
	When I go shopping, I often use bags that I bring from home.	1 = almost never or never; 5 = almost always or always
	I avoid battery-powered devices.	1 = almost never or never; 5 = almost always or always
**Recycling**	I recycle or use returnable bottles, cans, paper and plastic packaging.	1 = almost never or never; 5 = almost always or always
	I bring empty glass bottles to a recycling bin.	1 = almost never or never; 5 = almost always or always
	I separate waste.	1 = almost never or never; 5 = almost always or always
	I keep gift wrapping paper for reuse.	1 = almost never or never; 5 = almost always or always
	For making notes, I take paper that is already used on one side.	1 = almost never or never; 5 = almost always or always
	I put empty batteries in the garbage. (R)	1 = almost never or never; 5 = almost always or always
**Consumer behaviour**	I or people close to me buy less stuff for myself to reduce global climate change.	1 = almost never or never; 5 = almost always or always
	I or my family buy organic food.	1 = almost never or never; 5 = almost always or always
	I use/ we use chemical substances against insects at home (e.g. mosquito sprays such as Autan, etc.). (R)	1 = almost never or never; 5 = almost always or always
	I eat seasonal produce.	1 = almost never or never; 5 = almost always or always
	When shopping, I prefer products with eco-labels.	1 = almost never or never; 5 = almost always or always
	I eat in fast-food restaurants, such as McDonalds and Burger King. (R)	1 = almost never or never; 5 = almost always or always

		I use writing pads from recycled paper.	1 = almost never or never; 5 = almost always or always
		I prefer markers to crayons for drawing. (R)	1 = almost never or never; 5 = almost always or always
		I avoid eating pre-packaged snacks/drinks with disposable packaging.	1 = almost never or never; 5 = almost always or always
		I order take-out pizza. (R)	1 = almost never or never; 5 = almost always or always
	**Indirect pro-environmental behaviour**	I use/ we use energy-efficient electronic appliances at home.	1 = almost never or never; 5 = almost always or always
		I buy/ we buy locally produces food at home.	1 = almost never or never; 5 = almost always or always
		I buy/ we buy seasonal fruit and vegetables.	1 = almost never or never; 5 = almost always or always
		I contribute/ our family contributes financially to environmental organizations.	1 = almost never or never; 5 = almost always or always
		I have pointed out unecological behaviour to someone.	1 = almost never or never; 5 = almost always or always
		I read books, publications, and other materials about environmental problems.	1 = almost never or never; 5 = almost always or always
		I learn about environmental issues in the media (newspapers, magazines, and TV).	1 = almost never or never; 5 = almost always or always
		We usually spend our holidays abroad. (R)	1 = almost never or never; 5 = almost always or always
		After a picnic, I leave the place as clean as it was before.	1 = almost never or never; 5 = almost always or always
Outcome variables	Meat intake	Choose the answer that best describes your eating habits. I eat…	1 = meat at most meals; 2 = meat at some meals, 3 = meat very rarely, 4 = no meat but fish, 5 = vegetarian, 6 = vegan

Past meat reduction	Now we want to know whether the frequency of meat consumption (including red meat, processed meat, poultry, fish, and seafood) has changed for you personally over the last three years?	1 = I have not eaten meat over a three-year period; 7 = I have increased the amount of meat in my diet considerably
Intention to reduce meat	Please think about the period of the next month. Do you think that the frequency of meat consumption (including red meat, processed meat, poultry, fish, and seafood) will change for you personally over the next month?	1 = I will continue to eat no meat; 7 = I will increase the amount of meat in my diet considerably

Notes. Answers in bold present the correct answer on question. Items which were reversed are marked with (R).
